# Protective RBD-dimer vaccines against SARS-CoV-2 and its variants produced in glycoengineered *Pichia pastoris*

**DOI:** 10.1371/journal.ppat.1012487

**Published:** 2024-08-30

**Authors:** Tongxin Zhao, Sheng Liu, Pengyan Wang, Yanfang Zhang, Xinrui Kang, Xiaoqian Pan, Linjie Li, Dedong Li, Ping Gao, Yaling An, Hao Song, Kefang Liu, Jianxun Qi, Xin Zhao, Lianpan Dai, Peipei Liu, Peiyi Wang, Guizhen Wu, Taicheng Zhu, Kun Xu, Yin Li, George F. Gao

**Affiliations:** 1 CAS Key Laboratory of Pathogen Microbiology and Immunology, Institute of Microbiology, Chinese Academy of Sciences (CAS), Beijing, China; 2 Cryo-EM Center, Southern University of Science and Technology, Shenzhen, China; 3 Medical School, University of Chinese Academy of Sciences (UCAS), Beijing, China; 4 Research Network of Immunity and Health (RNIH), Beijing Institutes of Life Science, Chinese Academy of Sciences (CAS), Beijing, China; 5 NHC Key Laboratory of Biosafety, National Institute for Viral Disease Control and Prevention, Chinese Center for Disease Control and Prevention, Beijing, China; 6 Department of Microbial Physiological & Metabolic Engineering, State Key Laboratory of Microbial Resources, Institute of Microbiology, Chinese Academy of Sciences (CAS), Beijing, China; University of Texas Medical Branch / Galveston National Laboratory, UNITED STATES OF AMERICA

## Abstract

Protective vaccines are crucial for preventing and controlling coronavirus disease 2019 (COVID-19). Updated vaccines are needed to confront the continuously evolving and circulating severe acute respiratory syndrome coronavirus 2 (SARS-CoV-2) variants. These vaccines should be safe, effective, amenable to easily scalable production, and affordable. Previously, we developed receptor binding domain (RBD) dimer-based protein subunit vaccines (ZF2001 and updated vaccines) in mammalian cells. In this study, we explored a strategy for producing RBD-dimer immunogens in *Pichia pastoris*. We found that wild-type *P*. *pastoris* produced hyperglycosylated RBD-dimer protein containing four N-glycosylation sites in *P*. *pastoris*. Therefore, we engineered the wild type *P*. *pastoris* (GS strain) into GSΔOCH1pAO by deleting the *OCH1* gene (encoding α-1,6-mannosyltransferase enzyme) to decrease glycosylation, as well as by overexpressing the *HIS4* gene (encoding histidine dehydrogenase) to increase histidine synthesis for better growth. In addition, RBD-dimer protein was truncated to remove the R328/F329 cleavage sites in *P*. *pastoris*. Several homogeneous RBD-dimer proteins were produced in the GSΔOCH1pAO strain, demonstrating the feasibility of using the *P*. *pastoris* expression system. We further resolved the cryo-EM structure of prototype-Beta RBD-dimer complexed with the neutralizing antibody CB6 to reveal the completely exposed immune epitopes of the RBDs. In a murine model, we demonstrated that the yeast-produced RBD-dimer induces robust and protective antibody responses, which is suitable for boosting immunization. This study developed the yeast system for producing SARS-CoV-2 RBD-dimer immunogens, providing a promising platform and pipeline for the future continuous updating and production of SARS-CoV-2 vaccines.

## Author summary

SARS-CoV-2 continuously circulates, resulting in infections and re-infections globally. COVID-19 vaccines can provide protection against disease and death caused by SARS-CoV-2. However, the immune responses induced by vaccination will wane gradually. In addition, new SARS-CoV-2 sub-variants emerge and show immune evasion. Therefore, inoculating with the updated COVID-19 vaccines is recommended for enhancing protection against the circulating SARS-CoV-2 sub-variants. *Pichia pastoris* is an eukaryotic system that enables fast and low-cost producing proteins with easy scale-up. In this study, we developed a strategy for producing SARS-CoV-2 RBD-dimer immunogens in glycoengineered *Pichia pastoris* with homogeneous glycosylation and high stability. The RBD antigen can be updated with the sequence of circulating sub-variants. In addition, mouse experiments demonstrated that the yeast-produced RBD-dimer vaccines induced robust and protective immune responses. This study provides a simple and promising approach to produce yeast-derived SARS-CoV-2 RBD-dimers as a boosting vaccine and guides the basis for the production of the other vaccines in *Pichia pastoris*.

## Introduction

Severe acute respiratory syndrome coronavirus 2 (SARS-CoV-2) remains a severe threat to global health, having caused almost 7 million deaths to date (https://covid19.who.int/). To combat the coronavirus disease 2019 (COVID-19) pandemic, vaccines have been developed using various platforms [1–3], including recombinant protein subunit vaccines[1], inactivated viruses vaccines[4], mRNA vaccines [5], DNA vaccines, and virus-vectored vaccines [6,7]. However, SARS-CoV-2 variants continuously emerge and circulate, escaping herd immunity. Additionally, the immune responses induced by vaccination and breakthrough infection will gradually wane [8,9]. Therefore, the administration of booster vaccines is crucial to enhance protection against the circulating SARS-CoV-2 variants, especially for the elderly, immunocompromised individuals, and people suffering from chronic diseases such as diabetes and hypertension.

SARS-CoV-2 spike (S) protein is responsible for recognizing the host cellular receptor ACE2 through its receptor-binding domain (RBD) [10]. Thus, the RBD is an attractive target to elicit neutralizing antibodies. Previously, we demonstrated that tandem-repeat RBD-dimers induced higher neutralizing antibody titers than traditional monomeric RBDs and developed the COVID-19 protein subunit vaccine ZF2001 [1,11]. Furthermore, we designed a heterologous RBD-dimer strategy to develop bivalent vaccines eliciting broad-spectrum immune responses [12]. RBD-dimer immunogens have also been used in mRNA, DNA and adenovirus-vector based vaccines to induce robust immune responses [13–16]. In the future, high-efficiency vaccines will likely be administered regularly like the current influenza vaccines. Thus, the COVID-19 vaccines should be safe, effective, easy to update, amenable to mass production, and low cost for use globally.

COVID-19 protein subunit vaccines have been successfully developed based on various immunogens [1,17,18], adjuvants [17,19–22] and expression systems [11,18,22]. Yeast expression systems, like *Pichia pastoris* [23], offer simple and cost-effective vaccine manufacturing and distribution platforms. They are anticipated to enhance global vaccine accessibility and equity, particularly for low- and middle-income countries (LMICs). Recent advancements have been made in expressing SARS-CoV-2 RBD antigens using *P*. *pastoris* [24–33]. In contrast to mammalian and insect cells, recombinant proteins secreted by the yeast cells are modified by hyperglycosylation at N-glycosylation sites. The published studies also report varying degrees of glycosylation of RBD protein produced by *P*. *pastoris* due to the N-glycosylation sites at N331 and N343 [29–31,34].

Hyperglycosylation can obscure immune epitopes and affect protective immune responses [35]. In addition, the lack of protein uniformity caused by hyperglycosylation complicates protein purification processes and potentially affects production consistency, thus raising concerns about the quality of large-scale production. To obtain the glycosylated RBD protein with better uniformity, researchers have attempted to mutate the N-glycosylation sites or use glycoengineered yeast as an expression host [24,27,32]. Among these studies, Corbevax [36] and Abdala [37] are based on the RBD monomer antigen produced in *P*. *pastoris* and have been demonstrated to be safe and immunogenic in phase III clinical trials. Zang *et al*. recently evaluated the immunogenicity of RBD-dimer produced in *P*. *pastoris* [31]. They found that RBD-dimer induced more potent immune responses than RBD monomer, consistent with our previous results from vaccines produced in mammalian cells [1]. However, these RBD-dimers encounter challenges with non-uniform glycosylation.

In this study, we explored the production of homogeneous RBD-dimer immunogens using the engineered *P*. *pastoris* system, analyzed the structure by cyro-EM, and evaluated their immunogenicity and protection efficacy in a murine model. This proof-of-concept study provides a strategy to produce bivalent COVID-19 RBD-dimer vaccine production in *P*. *pastoris*, contributing to the prevention of the continuously circulating SARS-CoV-2.

## Results

### Hyperglycosylation of prototype-Beta RBD-dimer protein produced in wild-type *P*. *pastoris*

**Fig 1:**
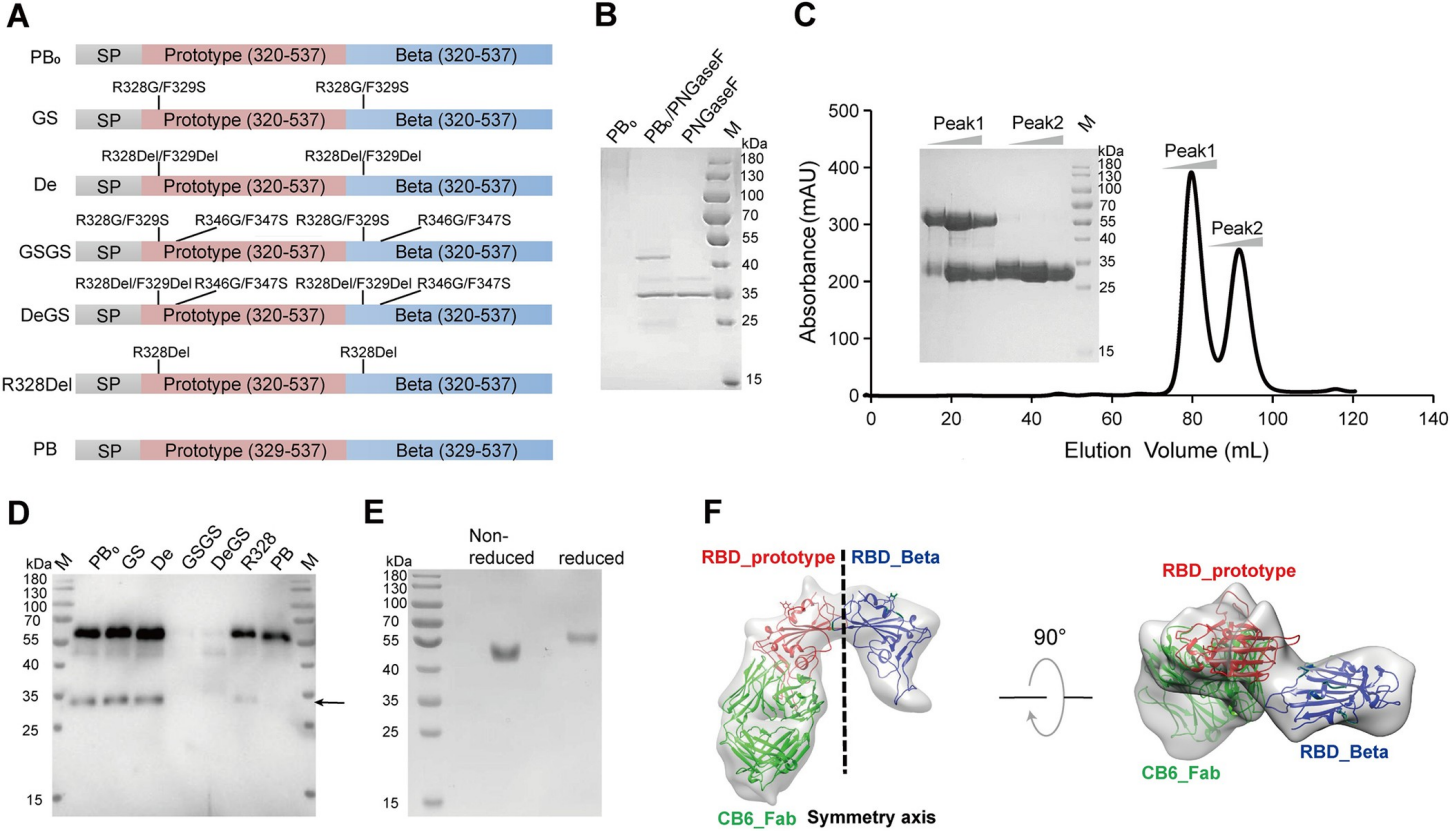
**Design of the RBD-dimers expressed in *P*. *pastoris*** A. Schematic diagram of the prototype-Beta RBD-dimer constructs. The prototype RBD (320–537) and Beta RBD (320–537) were dimerized tandemly and defined as PB_0_. Similarly, six further engineered prototype-Beta RBD-dimer constructs were designated as GS, De, GSGS, DeGS, R328-De, and PB, respectively. SP indicates signal peptide. B. SDS-PAGE analysis of the PB_0_ protein expressed in GS115. The PB_0_ protein was digested with PNGase F and then used for SDS-PAGE analysis. Lane PB_0_ showed the smeared PB_0_ protein; Lane PB_0_/PNGaseF showed the PB_0_ protein digested with PNGaseF. Lane PNGaseF showed the band of PNGaseF protein. C. Analytical gel filtration profile of the PB_0_ protein expressed in GSΔOCH1pAO. The gel filtration was performed with HiLoad 16/600 Superdex 200 pg. The 280-nm absorbance curves are shown. SDS-PAGE migration profiles of the elution are presented. The wedge symbols represent the sequential order of elution from gel filtration, clearly shown the co-existence of dimer and monomer in the expressed protein of PB_0_ design. D. Western blotting of supernatants from candidate yeast cultures. Anti-RBD polyclonal antibody was used as the primary probing antibody, clearly shown the broken-down monomer band as indicated by “→”. E. SDS-PAGE analysis of the PB protein produced in GSΔOCH1pAO. The SDS-PAGE migration profile of the PB protein is shown at non-reduced and reduced conditions, clearly shown the intact dimer without broken-down monomer with new PB design. F. Density map of prototype-Beta RBD-dimer bound to one CB6 Fab. Atomic models of the SARS-CoV-2 RBD (PDB: 6LZG) and the RBD/CB6 complex (PDB: 7C01) were fitted and rebuilt, clearly shown Beta RBD escaped the CB6 mAb.

Previously, we demonstrated the strategy of using RBD-heterodimers as bivalent vaccines to induce broad-spectrum immune responses [12]. Here, we attempted to prepare SARS-CoV-2 prototype-Beta (PB_0_) RBD-dimer protein using *P*. *pastoris*, in which the C-terminus of the prototype RBD was fused to the N-terminus of the Beta RBD (Fig 1A). The recombinant PB_0_ protein was expressed in the wild-type GS115 strain and purified from the culture supernatant. SDS-PAGE analysis revealed dispersed and non-homogenous PB_0_ protein on the gel (Fig 1B). After incubation of the PB_0_ protein with peptide-N4-(Nacetyl-beta-glucosaminyl) asparagine amidase (PNGaseF) and SDS-PAGE analysis, a distinct band emerged with a molecular weight of approximately 50 kDa (Fig 1B), which is the expected molecular weight of the PB_0_ protein. The other band indicated the PNGaseF protein (Fig 1B). This result suggested that the PB_0_ RBD-dimer protein expressed in *P*. *pastoris* contains severe N-glycan heterogeneity with hyperglycosylation from the wild-type GS115 strain.

### Development of the approaches for producing homogeneous RBD-dimer protein in *P*. *pastoris*

Subsequently, we focused on engineering the glycosylation process of the *P*. *pastoris* host. The α-1,6-mannosyltransferase enzyme, encoded by the gene *OCH1*, initiates the first step of the hyperglycosylation reaction in yeast [38]. We previously constructed a mutant *P*. *pastoris* strain GSΔOCH1 by knocking out the *OCH1* gene [39]. In addition, to restore histidine synthesis ability for better growth, the pAoα vector containing the *HIS4* gene encoding histidine dehydrogenase was further introduced into GSΔOCH1. This strain was named GSΔOCH1pAO. Then, we expressed the PB_0_ protein in the GSΔOCH1pAO strain and purified it. As expected, the purified PB_0_ exhibited a distinct band by SDS-PAGE analysis. Its molecular size was approximately 60 kDa (Fig 1C). However, gel filtration chromatography revealed that another protein with a molecular weight of ~30 kDa was also generated (Fig 1C). These results revealed that the glycoengineered strain GSΔOCH1pAO remarkably improved the uniformity of the PB_0_ protein produced compared to the wild-type strain and the expressed PB_0_ dimer protein was prone to digestion (Fig 1B and 1C).

To identify the potential cleavage sites on the PB_0_ dimer, we analyzed the protein sequence of the two distinct proteins (~60 and ~30 kDa) using N-terminal Edman degradation sequencing. We found that the N-terminal sequences of these two proteins were VQPTE (S 320–324) and FPNIT (S 329–333), respectively, suggesting the cleavage site, R328/F329, on the PB_0_ protein. Meanwhile, we found that another R346/F347 motifs on the PB_0_ protein and speculated that it was extra potential protease cleavage site.

We further designed six prototype-Beta RBD-dimer protein constructs to eliminate both R328/F329 and R346/F347 cleavage sites. The GS candidate contained two R328G and two F329S mutations. The De candidate was the RBD-dimer protein with deletion of R328 and F329. The GSGS and DeGS candidates contained another two R346G and F347S mutations compared to the GS and De proteins, respectively. The R328De candidate was only deleted for both R328 amino acids. The PB candidate contains two truncated RBDs (329–537) to remove the 328R/329F motif (Fig 1A). Next, we generated GSΔOCH1pAO strains to express these six RBD-dimer candidates, and the culture supernatants were harvested. The protein expression was analyzed using Western blotting. We found that the PB construct was the best design to eliminate cleavage among these six candidates (Fig 1D). Then, the PB protein was purified and analyzed by SDS-PAGE, showing a clear, single band of the correct molecular weight (Fig 1E). These findings indicated that the N-terminal truncation design effectively addresses the issue of RBD-dimer protein cleavage using the *P*. *pastoris* system and thus enabled us to obtain a stable PB dimer protein for further investigation.

Next, we resolved the cryo-EM structure of PB protein produced by GSΔOCH1pAO complexed with the fragment antigen binding (Fab) portion of antibody CB6. The rebuilt models showed their overall conformation (Fig 1F). The PB RBD-dimer was arranged as a bilateral lung-like structure with approximately axial symmetry and engaged one CB6 Fab at one arm, consistent with our previous study on PB protein produced in mammalian cells [12]. The structure demonstrated that the PB RBD-dimer produced in the *P*. *pastoris* GSΔOCH1pAO strain correctly exposed major immune epitopes (Fig 1F).

**Fig 2:**
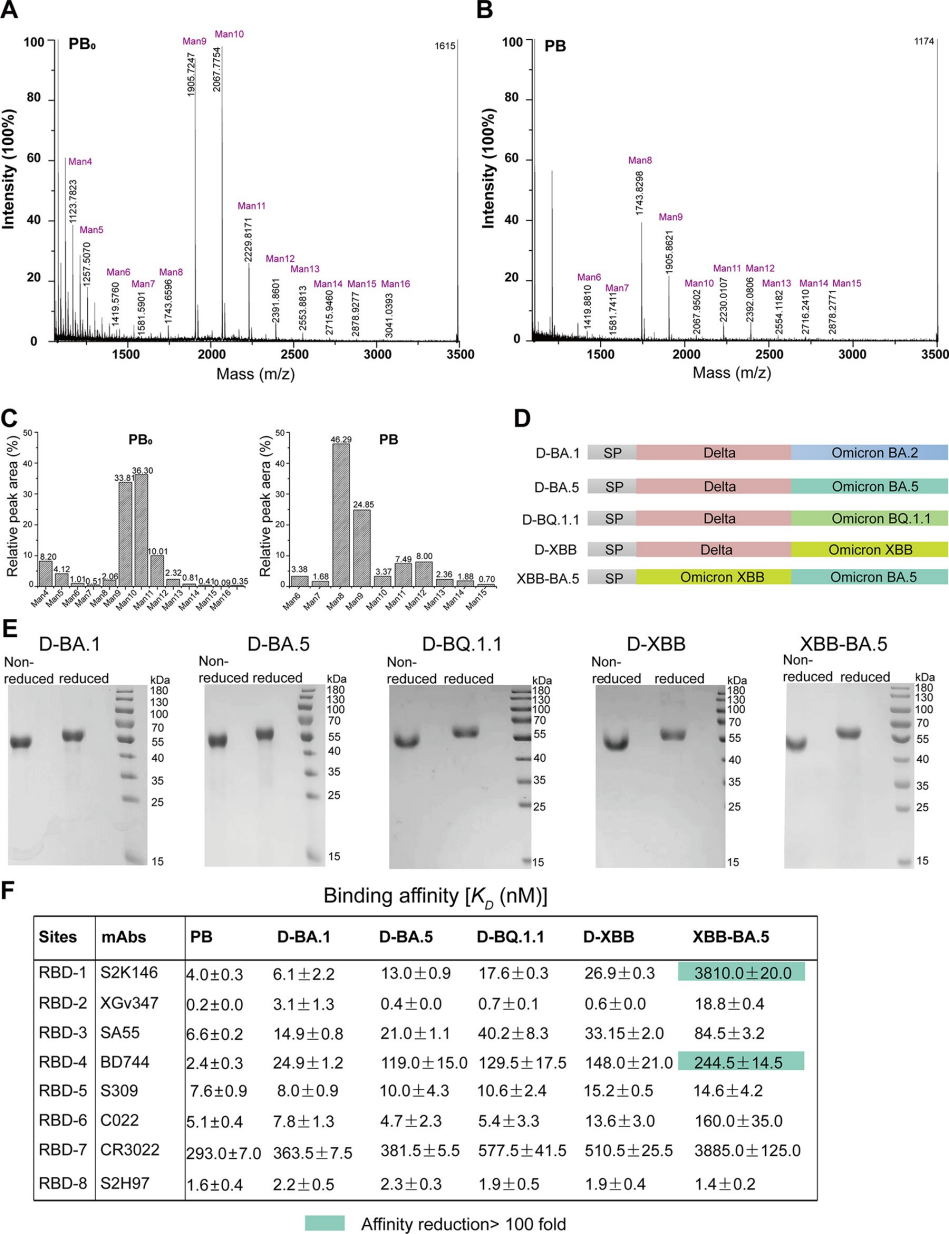
Characterization of RBD-dimers produced in *P*. *pastoris*. A. Chromatogram of MALDI-TOF mass spectrometry of glycans released from PB_0_ produced in *P*. *pastoris* GS-PB_0_. Man indicates mannose. B. Chromatogram of MALDI-TOF mass spectrometry of glycans released from PB produced in *P*. *pastoris* GSΔOCH1pAO-PB. Man indicates mannose. C. Relative peak areas of identified mannose structures cleaved off from the PB_0_ and PB RBD-dimers. D. Schematic diagram of Delta-Omicron and Omicron_1-Omicron_2 RBD-dimers. The truncated RBDs (329–537) were dimerized tandemly. Delta-Omicron RBD-dimers include Delta-Omicron BA.1 (D-BA.1), Delta-Omicron BA.5 (D-BA.5), Delta-Omicron BQ.1.1 (D-BQ.1.1) and Delta- Omicron XBB (D-XBB). Omicron_1-Omicron_2 indicates Omicron XBB-Omicron BA.5 (XBB-BA.5). E. SDS-PAGE analysis of purified RBD-dimers produced in GSΔOCH1pAO. The SDS-PAGE migration profiles of these RBD-dimers are shown at non-reduced and reduced conditions. F. Binding affinities of antigens bound to representative mAbs targeting eight major epitopes. Affinity reductions more than 100-fold are colored in green.

Subsequently, we analyzed the N-glycan modification of the RBD-dimers produced by the engineering strain GSΔOCH1pAO-PB or the wild-type GS-PB0 strain through Matrix-Assisted Laser Desorption/Ionization Time of Flight Mass Spectrometry (MALDI-TOF-MS). We found that the PB protein exhibited relatively more homogeneous glycosylation than the PB_0_ protein (Fig 2A and 2B). The glycopattern of PB_0_ contains several high-mannose structures ranging from Man4 to Man16, with the dominant glycans identified as Man9 and Man10. In contrast, the PB protein featured a narrower range of high-mannose structures, mainly from Man8 to Man12, with the dominant glycan being Man8 (Fig 2C). The relative peak areas clearly underlined this observation (Fig 2C). These results demonstrated that the engineered strain GSΔOCH1pAO could express glycoprotein with more homogeneous glycosylation.

### RBD heterodimers against emerging SARS-CoV-2 variants

Due to the continuously mutating and severe immune evasion of emerging SARS-CoV-2 variants, we designed and expressed a series of RBD-dimer immunogens including Delta-BA.1, Delta-BA.5, Delta-BQ.1.1, Delta-XBB, and XBB-BA.5 by the developed *P*. *pastoris* system (Fig 2D). Like the PB RBD-dimer, all these proteins were successfully obtained with high stability, homogeneity (Fig 2E). These findings demonstrated the versatility of this *P*. *pastoris* system to produce sequence-updated bivalent SARS-CoV-2 RBD-dimer antigens.

Next, we selected eight representative SARS-CoV-2 monoclonal antibodies (mAbs) targeting eight RBD epitopes, respectively, to measure their binding affinities to each RBD-dimer protein produced by *P*. *pastoris* (Fig 2F). We observed that all the tested RBD-dimer proteins showed similar binding affinities to S309 (RBD-5) and S2H97 (RBD-8), which are broad-reactive to SARS-CoV-2 sub-variants, indicating the exposure of these RBD epitopes (Fig 2F). However, the XBB-BA.5 RBD-dimer showed decreased binding affinities to the other epitopes compared to prototype-Beta RBD-dimer, especially for S2K146 (RBD-1) and BD744 (RBD-4) with more than 100-fold decrease (Fig 2F). XBB and BA.5 sub-variants partially escaped the binding and neutralization of S2K146 and BD744 [40, 41]. Therefore, we speculate that the mutations caused decreased binding affinities. Our data suggested that the major neutralizing epitopes of the RBD-dimer were exposed.

### Immunogenicity of the SARS-CoV-2 RBD-dimer produced by the GSΔOCH1pAO strain

**Fig 3:**
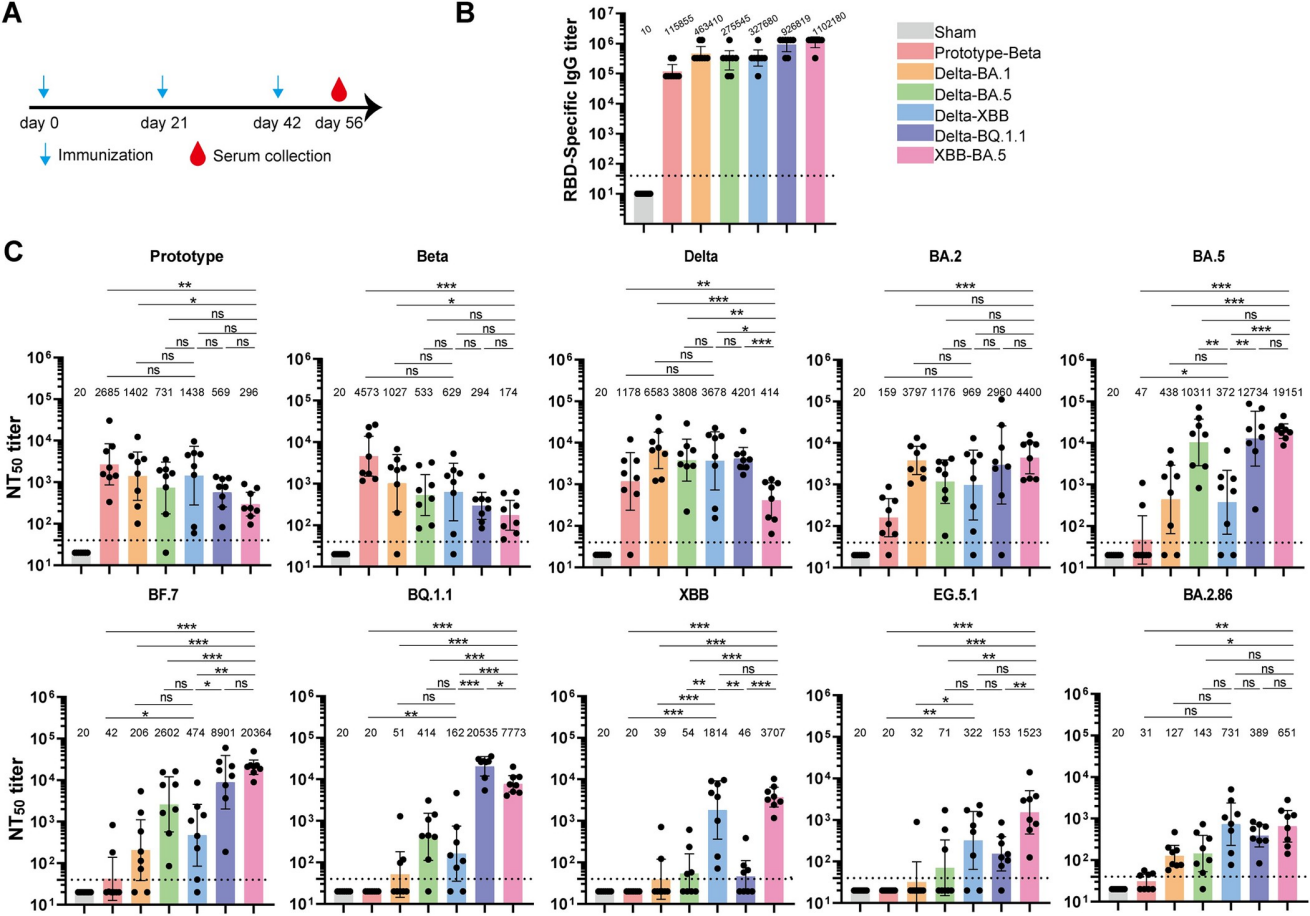
Antibody responses induced by various RBD-dimers in mice. A. Immunization schedule. Groups of 6- to 8-week-old female BALB/c mice (n = 8) were immunized with three doses of immunogen (2 μg) adjuvanted with AddaVax at days 0, 21 and 42. PBS plus adjuvant was given as the sham control. Murine sera were collected at day 56 for antibody titration. B. ELISA endpoint titers of antigen-binding IgG in murine sera. The respective RBD-dimer protein was coated for each group. C. Neutralization activity against SARS-CoV-2 pseudovirus of the murine sera. A panel of pseudoviruses displaying spikes from various SARS-CoV-2 variants, including prototype, Beta, Delta, Omicron BA.2, BA.5, BF.7, BQ.1.1, XBB, EG.5.1 and BA.2.86 were used. The values shown represent the GMT ± 95% confidence interval (CI). The horizontal dashed line represents the limit of detection (LOD). P values were analyzed with two-tailed Mann-Whitney tests (ns, p > 0.05; *p < 0.05; **p < 0.01; ***p < 0.001).

To analyze the immunogenicity of RBD-dimer vaccines, seven groups of BALB/c mice were immunized with three doses of each protein adjuvanted by AddaVax. Another group of BALB/c mice was immunized with PBS plus adjuvant as a sham control (Fig 3A). Murine sera were collected for antibody titrations. We found that the RBD-dimers potently elicited RBD-binding IgG, with the geometric mean titer (GMT) ranging from 115,855 to 1,102,180 (Fig 3B). Neutralizing antibody titers were tested using a panel of pseudotyped viruses displaying SARS-CoV-2 S protein (Fig 3C). Each RBD-dimer vaccine induced strong neutralizing responses against the antigen-match variants. However, we observed different cross-neutralizing profiles among these vaccines. Overall, the XBB-BA.5 RBD-dimer vaccine elicited the broadest neutralization spectra with pseudovirus neutralization titers (pVNT_50_) of 296, 174, 414, 4400, 19151, 20364, 7773, 3707, and 1523 against prototype, Beta, Delta, BA.2, BA.5, BF.7, BQ.1.1, XBB, and EG.5.1, respectively (Fig 3C). The cross-neutralizing responses against the currently emerging BA.2.86 variant were higher in the Delta-XBB (GMT: 731), Delta-BQ.1.1 (GMT: 389), and XBB-BA.5 (GMT: 651) groups (Fig 3C).

In addition, we collected the murine sera of the Delta-XBB and XBB-BA.5 RBD-dimer groups in the 22nd week to measure their neutralization activities. We found that the neutralizing antibody titers persisted at similar levels from the 8th to the 22nd week (S1 Fig).

### Antibody responses of booster vaccination of RBD-dimer vaccine after prototype vaccine priming

**Fig 4:**
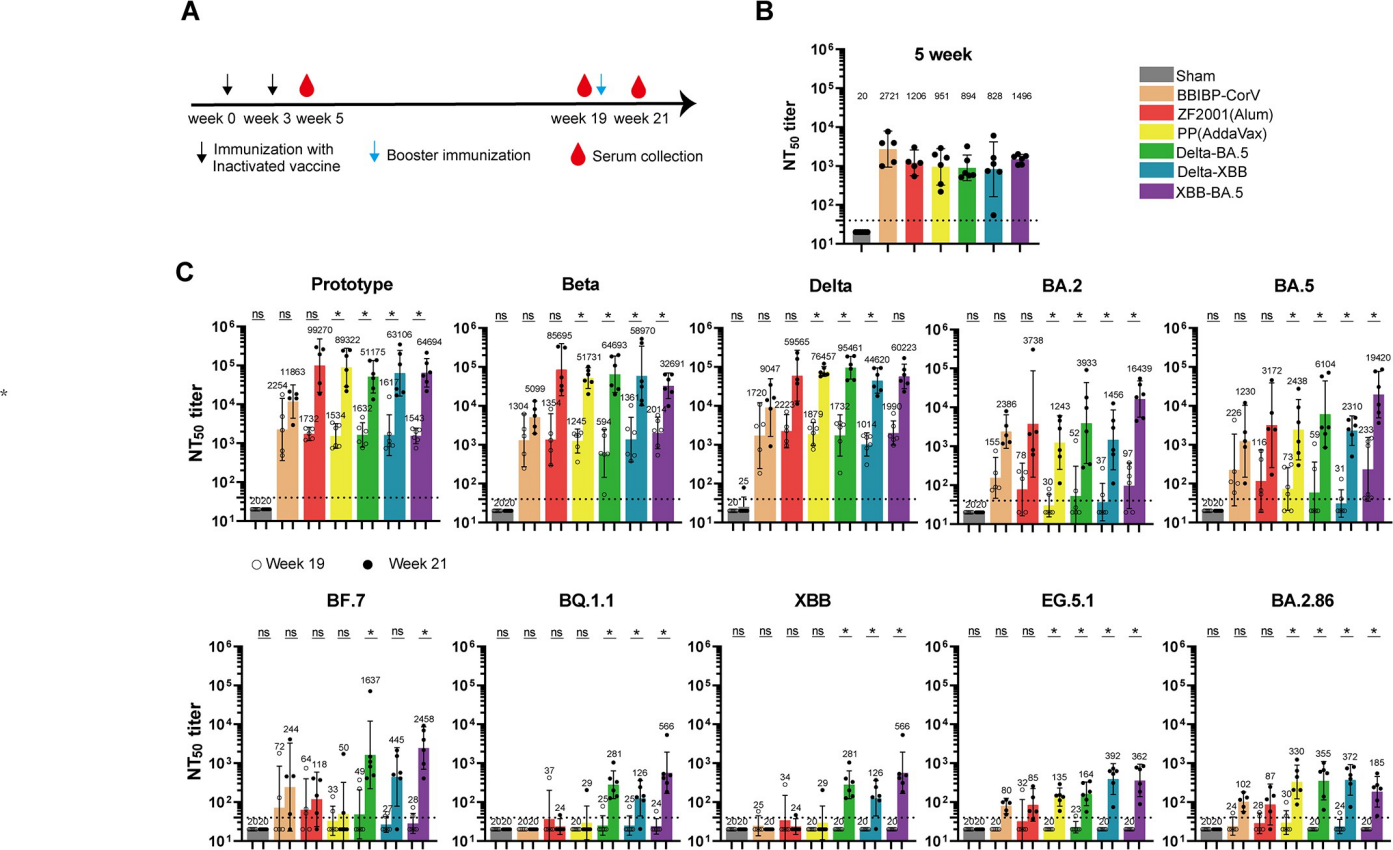
Immunogenicity of various RBD-dimers as booster vaccines. A. Immunization schedule. Groups of 6- to 8-week-old female BALB/c mice (n = 6) were immunized with two inactivated vaccines at weeks 0 and 3, followed by a booster dose of RBD-dimers adjuvanted with AddaVax at week 19. Three doses of PBS plus AddaVax were given as the sham control. Murine sera were collected at weeks 5, 19 and 21. B. Neutralization activity against prototype SARS-CoV-2 pseudovirus of murine sera collected at week 5. PP represents prototype RBD-homodimer. C. Neutralization activity against SARS-CoV-2 pseudoviruses of murine sera collected at weeks 19 and 21. A panel of pseudoviruses displaying prototype, Beta, Delta, Omicron BA.2, BA.5, BF.7, BQ.1.1, XBB, EG.5.1 or BA.2.86 spikes were used. The values shown represent the GMT ± 95% CI. The horizontal dashed line represents the LOD. P values were analyzed with Wilcoxon matched-pairs signed-rank tests (ns, p > 0.05; *p < 0.05).

Given that the COVID-19 inactivated virus vaccines were widely used in China and other countries [2,3], booster vaccination is needed to enhance the protection against circulating SARS-CoV-2 strains. Therefore, we evaluated the effect of Delta-BA.5, Delta-XBB, and XBB-BA.5 RBD-dimers as booster vaccines in a murine model. The mice were immunized with two doses of inactivated virus vaccine BBIBP-CorV (Sinopharm) for priming at weeks 0 and 3, respectively (Fig 4A). The inactivated virus vaccine-elicited neutralizing antibody responses against prototype pseudovirus were detected and displayed similar titers for each group of mice (Fig 4B). The RBD-dimer immunogen adjuvanted with AddaVax was injected for boosting at week 19. Blood samples were collected 2 days before and 14 days after boosting (Fig 4A). We observed that the BBIBP-CorV or ZF2001 booster induced low or even undetectable neutralizing antibody responses against recently emerging and circulating strains, including BF.7, BQ.1.1, XBB, EG.5.1, and BA.2.86 (Fig 4C). In comparison, GMTs against BA.5 and its sub-variants (BF.7 and BQ.1.1) induced by the Delta-BA.5 and XBB-BA.5 vaccines were higher than the others. GMTs against XBB and its sub-variant EG.5.1 induced by the Delta-XBB and XBB-BA.5 vaccines were higher than the other vaccines (Fig 4C). PP, Delta-BA.5, and Delta-XBB induced similar GMTs (between 330 and 372) against BA.2.86. These results indicated that updated vaccines should be antigenically close to the circulating SARS-CoV-2 strains to enhance protection efficacy, and the *P*. *pastoris* system is suitable for producing updated RBD-dimer immunogen as a booster vaccine.

### Protection efficacy of RBD-dimer vaccine produced by *P*. *pastoris*

**Fig 5:**
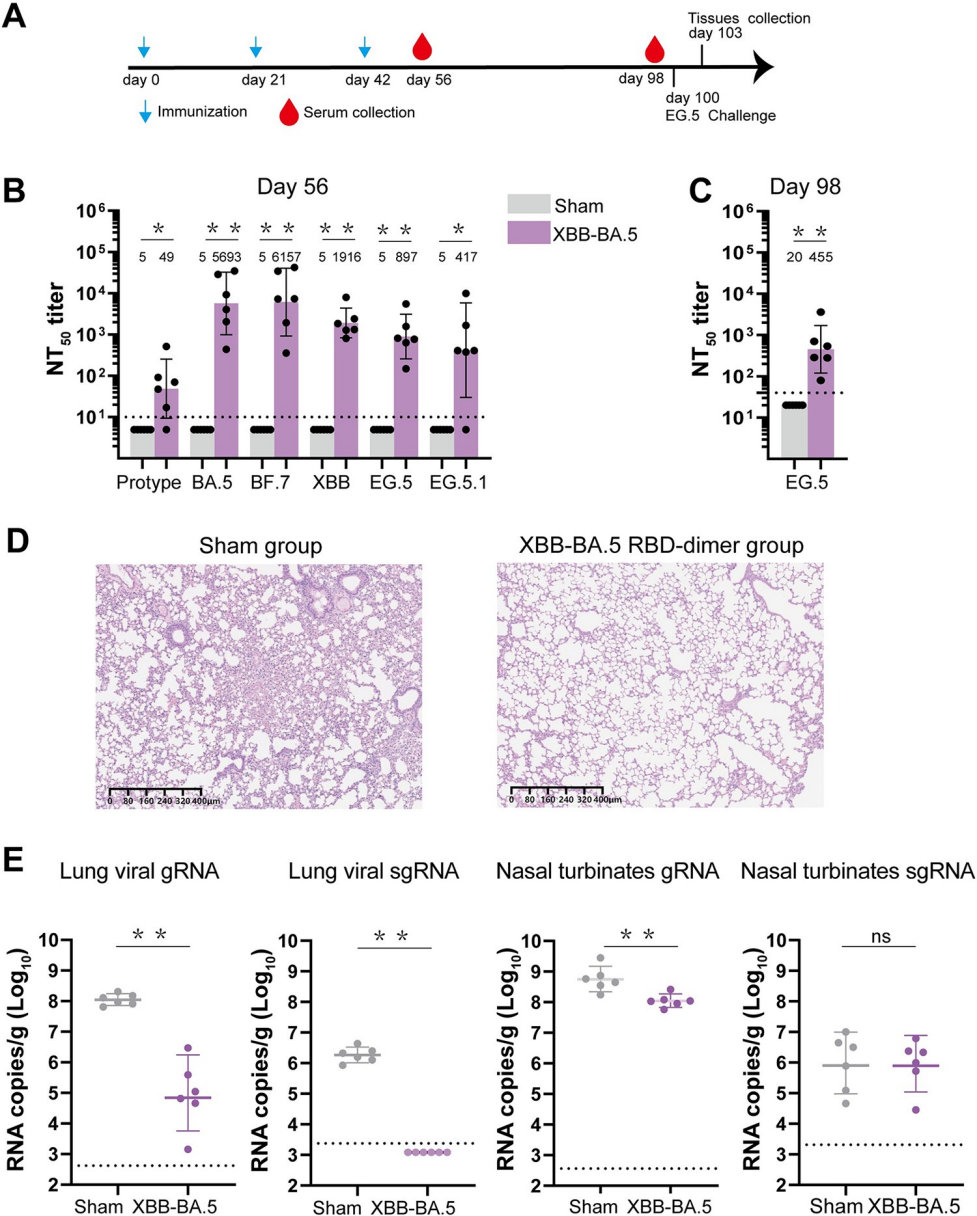
Protection efficacy of XBB-BA.5 RBD-dimer vaccine. A. Immunization and SARS-CoV-2 challenge schedule. Groups of 6- to 8-week-old female BALB/c mice (n = 6) were immunized with three doses of XBB-BA.5 RBD-dimer (2 μg) adjuvanted with AddaVax at days 0, 21 and 42. PBS plus adjuvant was given as the sham control. Murine sera were collected at days 56 and 97. Mice were intranasally infected with Omicron EG.5 via the intranasal route at days 100. B. Neutralization activity of murine sera collected at day 56. A panel of pseudoviruses displaying prototype, Omicron BA.5, BF.7, XBB, EG.5, EG.5.1 or BF.7 spikes were used. The values shown represent the GMT ± 95% CI. C. Neutralization activity against Omicron EG.5 pseudovirus of murine sera collected at day 98. The values shown represent the GMT ± 95% CI. D. Histological pathology of murine lung sections. E. Viral loads of murine lung and turbinates. The Omicron EG.5 gRNA and sgRNA levels were detected by qRT-PCR. The horizontal dashed line represents LOD. P values were analyzed with two-tailed Mann-Whitney tests (ns, p > 0.05; *p < 0.05; **p < 0.01).

To further analyze the protection efficacy of the RBD-dimer vaccine produced by *P*. *pastoris*, we conducted challenge experiments with the currently circulating SARS-CoV-2 variant EG.5 (Fig 5A). The mice were immunized three doses of XBB-BA.5 RBD-dimer vaccine or sham control, respectively (Fig 5A). Blood samples were collected on days 56 and tested for neutralization a panel of SARS-CoV-2 pseudoviruses (Fig 5B). Two days before the virus challenge, murine sera were collected and tested for neutralizing activities. The GMT against EG.5 in the XBB-BA.5 group was 455 (Fig 5C). The mice from the XBB-BA.5 and sham groups were intranasally challenged with the EG.5 strain at day 100. These mice were euthanized and necropsied on day 103 (Fig 5A). Lung tissues were collected and split into two portions for virus titration and pathological examination (Fig 5D). We quantified viral genomic (g)RNA and subgenomic (sg)RNA to assess viral replication in lungs and turbinates (Fig 5E). After being challenged with EG.5, mice from the sham group developed high levels of viral gRNA (1.12 x 10^8^ copies/g) and sgRNA (1.84 x 10^6^ copies/g) in the lungs (Fig 5E). In contrast, the XBB-BA.5 vaccine group exhibited significantly reduced lung viral gRNA (9.03 x 10^4^ copies/g) and undetectable lung viral sgRNA, indicating the complete control of viral replication in the lungs (Fig 5E). Compared to the high levels of the nasal turbinates gRNA (5.69 x 10^8^ copies/g) detected in the sham group, the gRNA GMT of the XBB-BA.5 vaccine group was marginally reduced to 1.12 x 10^8^ copies/g (Fig 5E). The nasal turbinates sgRNA GMTs of the sham and XBB-BA.5 vaccine groups were similar (~10^6^ copies/g) (Fig 5E). These results indicated that the protection against viral infection in nose provided by the vaccine is limited (Fig 5E). In addition, the lung sections were stained by hematoxylin and eosin (H&E) and analyzed for histopathology (Fig 5D). The murine lungs in the sham group showed moderate-to-severe histopathological changes (Fig 5D). In comparison, a significantly reduced lung injury and inflammatory response were seen in the mice vaccinated with the XBB-BA.5 RBD-dimer (Fig 5D). Therefore, these results demonstrated that the RBD-dimer vaccine derived from *P*. *pastoris* provides robust protection against SARS-CoV-2.

## Discussion

In this study, we expressed the SARS-CoV-2 RBD heterodimer protein in wild-type *P*. *pastoris* and found that the RBD-dimer protein contained heterogeneous hyperglycosylation. Therefore, we engineered *P*. *pastoris* by knocking out the *OCH1* gene to reduce glycosylation and to overexpress the *HIS4* gene to restore histidine synthesis ability for compensating yeast growth. The RBD-dimer construct was also truncated (S protein 329–537) to eliminate the 328R/329F cleavage motifs in the *P*. *pastoris*. Eventually, we obtained RBD-dimer proteins with high purity using the engineered *P*. *pastoris* strain GSΔOCH1pAO, demonstrating the efficiency of this *P*. *pastoris* expression system. We resolved the cryo-EM structure of the *P*. *pastoris*-produced PB RBD-dimer protein complexed with the CB6 Fab, revealing the correct protein folding and the fully exposed immune epitopes. Mouse experiments demonstrated that the *P*. *pastoris*-produced RBD-dimer vaccines induced robust and protective immune responses against SARS-CoV-2.

Compared to glycosylation patterns of mammalian cells, yeast-derived secreted proteins appear to have hypermannosylated N-glycans [42]. Previous studies reported similar heterogeneity in RBD monomers with two N-glycosylation modification sites produced in *P*. *pastoris* [29]. Two measurements have been taken to address the heterogeneity of secreted proteins in *P*. *pastoris*. One is to delete the glycosylation modification sites. The other one is to use glycoengineered yeast as a host. Because RBD-dimers have four N-glycosylation modification sites, it is better to use glycoengineered yeast as a host. In 2003, humanized N-linked glycosylation in *P*. *pastoris* was enabled by deleting eight endogenous genes and introducing six heterologous genes [43]. However, constructing glycoengineered yeast is time-consuming and labor-intensive, making it challenging to use widely. In *P*. *pastoris*, the alpha-1,6-mannosyltransferase encoded by the *OCH1* gene triggers additional mannosylation on the core glycoside modification. To prevent the hypermannosylated N-glycan modification, several secreted proteins were homogeneously expressed in an engineering strain with *OCH1* knockout [44–46]. Similarly, we used the GSΔOCH1pAO strain as the host to express RBD-dimers, achieving more homogeneously glycosylated protein preparations in our work.

In the mouse challenge experiment, the turbinates viral gRNA titer of the XBB-BA.5 RBD-dimer group marginally decreased compared to the sham control group, though statistical analysis indicated a significant difference. The viral sgRNA titer of these two groups showed similar levels, indicating that this vaccine provided much-limited protection against nasal viral infection. This is consistent with the observations from real-world, where COVID-19 vaccines administered intramuscularly prevent illness and death but do not effectively prevent viral infection in the upper respiratory tract. In addition, a small number of animals per group to evaluate their immunogenicity and protection efficacy. This is a limitation of this study.

The protein subunit vaccine ZF2001 (RBD homodimer protein adjuvated by aluminum hydroxide) is safe and effective against COVID-19, which has been approved for use since March 2021 [11]. The updated vaccines of ZF2001 contain RBD heterodimer immunogens and induce broad immune responses [12,47]. In addition, the other studies revealed that the S2P protein vaccine and ferritin nanoparticle vaccines displaying RBD did not elicit higher neutralizing antibody responses than the RBD-dimer protein [48, 49]. Our previous study showed that mosaic nanoparticles could elicit more antibodies targeting the conserved RBD epitopes with broad-spectrum activities [50]. Therefore, we speculated that the RBD heterodimer may better stimulate these conserved antigenic epitopes than the RBD homodimer, In-depth mechanisms should be further studied. Compared to mammalian cells (CHO cells), which are used for producing the RBD-dimer protein of ZF2001, *P*. *pastoris* is an alternative eukaryotic system that enables faster and low-cost process development with easy scale-up especially used in LMICs [51]. Several biopharmaceutical products produced by *P*. *pastoris* have been approved for human use, including Gavac [52], Shanvac [53] and Kalbitor [54].

SARS-CoV-2 continuously evolves and circulates globally. The cross-neutralization activities induced by the RBD-dimers were limited due to the continuous evolution and emergence of SARS-CoV-2 sub-variants. According to the recommendations raised by the WHO Technical Advisory Group on COVID-19 Vaccine Composition (TAG-CO-VAC), updating the immunogen sequence is necessary to enhance efficacy against recently circulating. This study provides approaches to produce yeast-derived SARS-CoV-2 RBD-dimers as a boosting vaccine to enhance the protection efficacy against circulating SARS-CoV-2 strains, which is a simple, fast and promising approach.

## Materials and methods

### Ethics statement

Animal studies were approved by the Committee on the Ethics of Animal Experiments of the Institute of Microbiology, Chinese Academy of Sciences (IMCAS)(SQIMCAS2022088), and performed in compliance with the recommendations in the Guide for the Care and Use of Laboratory Animals of the IMCAS Ethics Committee. The mice challenge experiments were conducted under ABSL3 facility in Chinese Center for Disease Control and Prevention, which were approved by the Ethics Committee of the National Institute for Viral Disease Control and Prevention, Chinese Center for Disease Control and Prevention (No. 20230914097).

### Construction and culture of *P*. *pastoris* strains

A list of the *P*. *pastoris* strains and plasmids used in this study is detailed in S1 and S2 Tables. All *P*. *pastoris* strains were constructed based on the GS115 or the mutant GS115ΔOCH1 [39]. In this study, the pAOαM plasmid was linearized using the SalI restriction enzyme and then electroporated into *P*. *pastoris* GS115OCH1. After selecting positive transformants on the MD plate, GSΔOCH1pAO was obtained. The genes encoding SARS-CoV-2 RBD-dimers were codon optimized, synthesized, and cloned into pMPICZα using the XhoI and NotI restriction sites (GenScript). The recombinant plasmids were linearized with SacI restriction enzyme and introduced into *P*. *pastoris* GS115 or GS115ΔOCH1pAO. Transformants were incubated on YPD plates containing 120 μg/mL Zeocin at 30°C for 48 h. Randomly selected colonies were evaluated for RBD-dimer protein expression using a BMMY medium with 0.5%-0.8% methanol. The RBD-dimer protein was identified by western blot with anti-RBD polyclonal antibodies.

### The expression and purification of RBD-dimer protein

Recombinant *P*. *pastoris* strains were cultured on a 1-liter fermentor (Infors, Switzerland). A 50 mL seed culture was inoculated into 0.8 L of BMGY medium. The culture was then incubated at 30°C, pH 6.0, and agitated at 600–900 rpm with an airflow of 2 vvm until the glycerol was depleted. After starvation for 1.5 h, the induction was initiated with 0.3% methanol under 25°C. A methanol feeding phase was started with a methanol concentration of 0.3%-0.5% in the medium, monitored and controlled by a methanol detection flow plus controller for approximately 70–90 hours. The fermentation supernatant was then collected by centrifuging.

The RBD-dimer proteins were purified from the supernatant through Ni affinity chromatography using a HisTrap HP 5 mL column (GE Healthcare, USA). The following purification step was performed with gel filtration chromatography using either a HiLoad 10/300 Superdex 200 pg (GE Healthcare, USA) or HiLoad 16/600 Superdex 200 pg (GE Healthcare, USA). The purified RBD-dimers were stored in PBS buffer (pH 7.4) containing 137 mM NaCl, 2.7 mM KCl, 10 mM Na_2_HPO_4_ and 2 mM KH_2_PO_4_. SDS-PAGE analysis was performed to measure the molecular size and purity of the purified protein. The fragment of antibody-binding (Fab) was prepared as previously described [12].

### Analysis of the N-glycans of RBD-dimers

The N-glycans from the RBD-dimers were analyzed by MALDI-TOF-MS [55]. Briefly, the N-glycans were released by incubating the proteins with Peptide-N4-(N-acetyl-β-D-glucosaminyl) asparigineamidase (PNGase F) overnight at 37°C. The N-glycans were then extracted using a solid-phase method based on porous graphitic carbon and then derivatized with formic amide. Purification was carried out using a solid-phase extraction method based on hydrophilic chromatography [56]. Finally, the purified N-glycans were analyzed by MALDI-TOF (AB SCIEX, USA) mass spectra in reflected positive ion mode. The N-glycans were dissolved in a 50% acetonitrile solution. To ionize the glycan as a sodium adduct, a 0.5 μL sample was dropped on an Anchor Chip MALDI target plate, followed by the addition of an equal volume of 10 mg/mL DHB matrix containing 10 mM sodium acetate anhydrous. Mass spectra, ranging from 1,000 to 4,000 m/z, were acquired in 1,000 shots at a 2,000 Hz laser frequency. The spectra were further processed by Data Explorer software (Applied Biosystems) with baseline correction, noise filtering and peak deisotoping [56].

### Cryo-EM data collection and 3D reconstruction

The PB/CB6 Fab dataset was processed by modifying the previously reported method [12]. Briefly, a total of 344,856 automatically picked particles were initially extracted in Relion. Then, the heterogeneous particles were removed by three rounds of reference-free 2D classification. Three rounds of 3D classification without applying symmetry were performed using a clean dataset consisting of 199,469 particles. Last, a density map at 15Å resolution was calculated by employing a 3D refinement with C2 symmetry after identifying a single dominant class (19,756 particles). Subsequently, the crystal structure of the PB/CB6 complex (PDB: 7C01) or its RBD part was fitted into the two density maps using CHIMERA [57] with high level of matching.

### Surface plasmon resonance (SPR) assay

The SPR assays were carried out using a BIAcore 8k (Cytiva) with single cycle method at 25°C. The PBST buffer (10 mM Na_2_HPO_4_, 2 mM KH_2_PO_4_ pH 7.4, 137 mM NaCl, 2.7 mM KCl, 0.05% Tween20) was used for kinetic analyses. RBD-dimers (including PB, D-BA.1,D-BA.5, D-BQ.1.1, D-XBB, XBB-BA.5) were diluted with sodium acetate pH 5.0 and immobilized on a CM5 chip with the standard EDC/NHS coupling method. Serially diluted antibody’s fab proteins flowed over the chip surface. Glycine (10 mM, pH 1.5) was used to regenerate the chips. The binding affinities (*K*_D_) were obtained using 1:1 binding model with the BIAcore 8K Evaluation software (GE Healthcare).

### Mouse experiments

RBD-dimer proteins were adjuvanted by AddaVax (InvivoGen, USA). Specific pathogen-free (SPF) female BALB/c mice were intramuscularly immunized with RBD-dimer vaccines (2 μg). The experimental schedules were indicated within the figure legends. The Omicron EG.5 challenge studies were conducted within an animal biosafety level 3 (ABSL3) facility in Chinese Center for Disease Control and Prevention (China CDC).

### Enzyme-linked immunosorbent assay (ELISA)

ELISAs were performed as previously described [58]. Briefly, 96-well plates (3590, Corning, USA) were coated overnight with 3 μg/mL of antigen proteins in 0.05 M carbonate-bicarbonate buffer (pH 9.6). The plates were blocked in 5% skim milk in PBS. Serum samples from mice were serially diluted and added to each well. The plates were incubated for 2 h and washed. Then, the plates were incubated with goat anti-mouse IgG-HRP antibody (Easybio, BE0102-100) for 1 h and then washed. The plates were subsequently developed with 3,3’,5,5’-tetramethylbenzidine (TMB) substrate. Reactions were stopped with 2 M hydrochloric acid, and the absorbance was measured at 450 nm using a microplate reader (Multiskan FC, Thermo Fisher). The endpoint titers were defined as the highest reciprocal dilution of serum to give an absorbance greater than 2.5-fold of the background values. Antibody titer below the limit of detection was determined as half the limit of detection.

### Pseudotyped virus neutralization assay

The pseudotyped viruses were prepared as previously described [59–62]. Serially diluted serum and pseudoviruses were mixed and incubated at 37°C for 1 h. The mixtures were then transferred to pre-plated Vero cell monolayers in 96-well plates, incubating for 15 h. The transducing unit values were recorded using a CQ1 confocal image cytometer (Yokogawa). Fifty percent of the pseudovirus neutralization titer (pVNT_50_) was determined by fitting nonlinear regression curves using GraphPad Prism and calculating the reciprocal of the serum dilution required for 50% neutralization of infection. pVNT_50_ below the limit of detection was determined as half the limit of detection.

### Challenge of mice with SARS-CoV-2 Omicron EG.5

The protective efficacy of RBD-dimer vaccine produced in *P*. *pastoris* was evaluated by challenge experiments. Mice were intranasally challenged with 2.5×10^4^ TCID_50_ of Omicron EG.5 (NPRC 2.192100031). A schedule was shown in Fig 5A. Three days post-infection, the mice were euthanized and necropsied. Murine lung tissues and turbinates were collected. Lung tissues were split into two parts for virus titration and pathological examination (hematoxylin and eosin staining). Virus titers were measured as previously described [12]. The levels of genomic RNA (gRNA) and subgenomic RNA (sgRNA) were analyzed by quantitative reverse transcription-PCR (qRT-PCR) assays, which were performed using a FastKing One Step Probe RT-qPCR kit (Tiangen Biotech, China) on a CFX96 Touch real-time PCR detection system (Bio-Rad, USA) according to the manufacturer’s protocol. The primers and probes were designed to target the N gene of the gRNA [63] and the E gene of sgRNA [64], with sequences as follows: gRNA-F, GACCCCAAAACCAGCGAAAT; gRNA-probe, ACTCCGCATTACGTTTGGTGGACC; gRNA-R, TCTGGTTACTGCCAGTTGAATCTG; sgRNA-F, TGATCTCTTGTAGATCTGTTCTC; sgRNA-probe, ACACTAGCCATCCTTACTGCGCTTCG; sgRNA-R, ATATTGCAGCAGTACGCACACA.

### Statistical analysis

Pseudovirus neutralization titers were determined by fitting nonlinear regression curves using GraphPad Prism and calculating the reciprocal of the serum dilution required for 50% neutralization of infection. The values are the GMT ± 95% confidence interval (CI). Details are shown in figure legends. P-values were analyzed with two-tailed Mann Whitney test or Wilcoxon matched-pairs signed rank test (ns, p > 0.05; *p < 0.05; **p < 0.01; ***p <0.001). Details are shown in figure legends.

## Supporting information

S1 Fig Long-term antibody responses induced by RBD-dimer vaccines. A. Immunization schedule. Groups of 6- to 8-week-old female BALB/c mice (n = 8) were immunized with three doses of immunogen (2 μg) adjuvanted with AddaVax at weeks 0, 3 and 6. PBS plus adjuvant was given as the sham control. Murine sera were collected at weeks 8 and 22. B. Detection of neutralization activity of murine sera. A panel of pseudoviruses displaying prototype, Beta, Delta, Omicron sub-variants BA.2, BA.5, BF.7, BQ.1.1, XBB, EG.5.1 or BA.2.86 spikes were used. The values were shown as GMT ± 95% CI. The horizontal dashed line represents the LOD. P values were analyzed with Wilcoxon matched-pairs signed rank tests (ns, p > 0.05; *p < 0.05; **p < 0.01).
(TIF)

S1 Table Plasmids used in this study. (PDF)

S2 Table Strains used in this study. (PDF)
