# Supplementary figures and images for: Protective RBD-dimer vaccines against SARS-CoV-2 and its variants produced in glycoengineered Pichia pastoris
Source: PLoS Pathog. 2024 Aug 30;20(8):e1012487. doi: 10.1371/journal.ppat.1012487 (PMC11364227; doi:10.1371/journal.ppat.1012487)

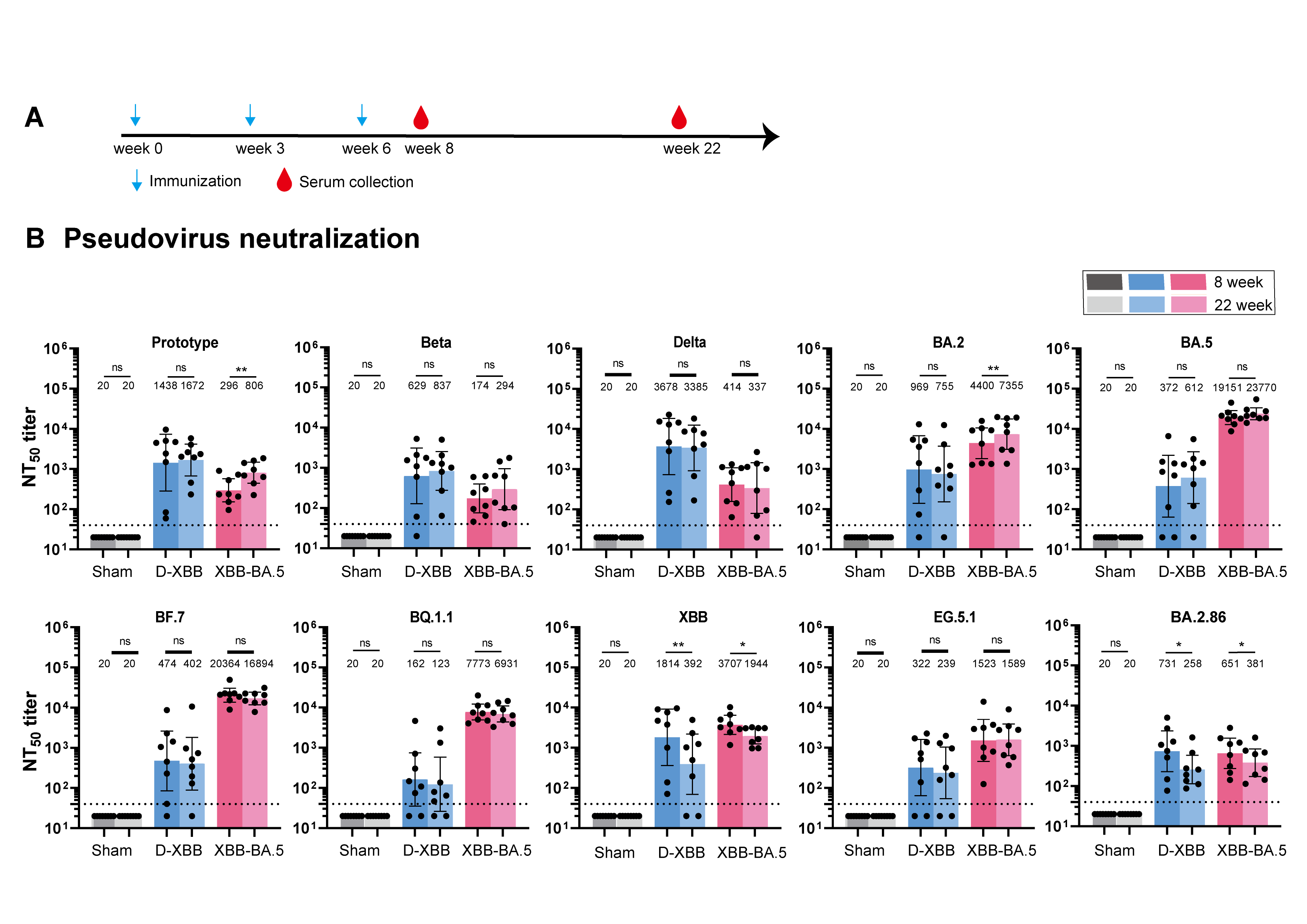

Supplement: S1 Fig — A. Immunization schedule. Groups of 6- to 8-week-old female BALB/c mice (n = 8) were immunized with three doses of immunogen (2 μg) adjuvanted with AddaVax at weeks 0, 3 and 6. PBS plus adjuvant was given as the sham control. Murine sera were collected at weeks 8 and 22. B. Detection of neutralization activity of murine sera. A panel of pseudoviruses displaying prototype, Beta, Delta, Omicron sub-variants BA.2, BA.5, BF.7, BQ.1.1, XBB, EG.5.1 or BA.2.86 spikes were used. The values were shown as GMT ± 95% CI. The horizontal dashed line represents the LOD. P values were analyzed with Wilcoxon matched-pairs signed rank tests (ns, p > 0.05; *p < 0.05; **p < 0.01). (TIF) [file ppat.1012487.s001.tif]
