# Supplementary material for: Protective RBD-dimer vaccines against SARS-CoV-2 and its variants produced in glycoengineered Pichia pastoris
Source: PLoS Pathog. 2024 Aug 30;20(8):e1012487. doi: 10.1371/journal.ppat.1012487 (PMC11364227; doi:10.1371/journal.ppat.1012487)
Supplement: S1 Table — (PDF) [file ppat.1012487.s002.pdf]

S1 Table Plasmids used in this study

Plasmids	Short descriptions	Reference or source
pAOαM	Vector for extracellular expression; containing *HIS4* gene	Invitrogen
pMPICZα	Vector for extracellular expression; derived from pPICZaA and pAO815, containing 6 His tag, zeo^r	In our lab
pMPICZα-PB_0-His	Recombinant Prototype-Beta chimeric RBD-dimer, spike residues 320-537 (Prototype, GenBank: YP_009724390) and 320-537 (Beta variant, GISAID: EPI_ISL_736940)	This study
pMPICZα-PB_0-His-GS	pMPICZα-PB_0-His vector with four mutant sites of R328G and F329S in each RBD	This study
pMPICZα-PB_0-His-De	pMPICZα-PB_0-His vector with four deletion sites of R328 and F329 in each RBD	This study
pMPICZαZα-PB_0-His-GSGS	pMPICZα-PB_0-His vector with eight mutant sites of R328G, F329S, R346G and R347S in each RBD	This study
pMPICZα-PB_0-His-DeGS	pMPICZα-PB_0-His vector with four deletion sites of R328G, F329S, and four mutant sites of R346G and R347S in each RBD	This study
pMPICZα-PB_0-His-R328De	pMPICZα-PB_0-His vector with two deletion sites of R328 in each RBD	This study
pMPICZα-PB-His	pMPICZα-PB_0-His vector with deletion of N-terminal 9 amino acids in each RBD	This study
pMPICZα-D-BA.1-His	Recombinant Delta-Omicron BA.1 chimeric RBD-dimer, spike residues 329-537 (Delta	This study

	variant, EPI_ISL_1758376), and 329-537 (Omicron BA.1 variant, EPI_ISL_18842611)	
pMPICZα-D-BA.5-His	Recombinant Delta-Omicron BA.5 chimeric RBD-dimer, spike residues 329-537 (Delta variant, EPI_ISL_1758376 and 329-537 (Omicron BA.5 variant, EPI_ISL_17636703)	This study
pMPICZα-D-BQ.1.1-His	Recombinant Delta-Omicron BQ.1.1 chimeric RBD-dimer, spike residues 329-537 (Delta variant, EPI_ISL_1758376 and 329-537 (BQ.1.1 variant, EPI_ISL_17636943)	This study
pMPICZα-D-XBB-His	Recombinant Delta-Omicron XBB chimeric RBD-dimer, spike residues 329-537 (Delta variant, EPI_ISL_1758376 and 329-537 (XBB variant, EPI_ISL_16392105)	This study
pMPICZα-XBB-BA.5-His	Recombinant Omicron XBB-Omicron BA.5 chimeric RBD-dimer, spike residues 329-537 (XBB variant, EPI_ISL_16392105 and 329-537 (BA.5 variant, EPI_ISL_17636703)	This study
