# Supplementary material for: Protective RBD-dimer vaccines against SARS-CoV-2 and its variants produced in glycoengineered Pichia pastoris
Source: PLoS Pathog. 2024 Aug 30;20(8):e1012487. doi: 10.1371/journal.ppat.1012487 (PMC11364227; doi:10.1371/journal.ppat.1012487)
Supplement: S2 Table — (PDF) [file ppat.1012487.s003.pdf]

S2 Table Strains used in this study

Strains	Short descriptions	Reference or source
E. coli DH 5a	Commercial transformation host for cloning	Tsingke
P. pastoris GS115	Commercial transformation host for Cloning; *HIS4*$^-$, Mut$^+$	Invitrogen
GS-PB$_0$	GS115 integrated with linearized pMPICZα-PB(320-537)-His	This study
GSΔOCH1	GS115 with its *OCH1* gene disrupted	In our lab
GSΔOCH1pAO	GS115ΔOCH1 integrated with linearized pAOαM; His$^+$	This study
GSΔOCH1pAO-PB$_0$	GS115ΔOCH1pAO integrated with linearized pMPICZα-PB$_0$-His	This study
GSΔOCH1pAO-GS	GS115ΔOCH1pAO integrated with linearized pMPICZα-PB$_0$-His-GS	This study
GSΔOCH1pAO-De	GS115ΔOCH1pAO integrated with linearized pMPICZα-PB$_0$-His-De	This study
GSΔOCH1pAO-GS GS	GS115ΔOCH1pAO integrated with linearized pMPICZα-PB$_0$-His-GSGS	This study
GSΔOCH1pAO-De GS	GS115ΔOCH1pAO integrated with linearized pMPICZα-PB$_0$-His-DeGS	This study
GSΔOCH1pAO-R32 8De	GS115ΔOCH1pAO integrated with linearized pMPICZα-PB$_0$-His-R328De	This study
GSΔOCH1pAO-PB	GS115ΔOCH1pAO integrated with linearized pMPICZα-PB-His	This study
GSΔOCH1pAO-D-B A.1	GS115ΔOCH1pAO integrated with linearized pMPICZα-D-BA.1-His	This study

GSΔOCH1pAO-D-BA.5	GS115ΔOCH1pAO integrated with linearized pMPICZα-D-BA.5-His	This study
GSΔOCH1pAO-D-BQ.1.1	GS115ΔOCH1pAO integrated with linearized pMPICZα-D-BQ.1.1-His	This study
GSΔOCH1pAO-D-XBB	GS115ΔOCH1pAO integrated with linearized pMPICZα-D-XBB-His	This study
GSΔOCH1pAO-XBB-BA.5	GS115ΔOCH1pAO integrated with linearized pMPICZα-XBB-BA.5-His	This study
